# Computer Based Body Exposure in Adolescents With Anorexia Nervosa: A Study Protocol

**DOI:** 10.3389/fpsyt.2021.769239

**Published:** 2022-01-24

**Authors:** Valeska Stonawski, Lena Sasse, Gunther Moll, Oliver Kratz, Stefanie Horndasch

**Affiliations:** Department of Child and Adolescent Psychiatry, Faculty of Medicine, Friedrich-Alexander-University Erlangen-Nürnberg, Erlangen, Germany

**Keywords:** anorexia nervosa, body dissatisfaction, body exposure, eye tracking, attentional bias, adolescence, stress reactivity, randomized controlled trial (RCT)

## Abstract

Body dissatisfaction is a core feature of eating disorders (EDs) and plays an essential role in the development and maintenance of anorexia nervosa (AN). In the current study, a computer based body exposure intervention is conducted and evaluated regarding short-term effects on body dissatisfaction, psychopathology, viewing patterns, and stress reactivity. Within a randomized controlled trial (RCT) female adolescents and young women with AN are either receiving the intervention or treatment as usual (TAU). Furthermore, in a transdiagnostic approach, a highly body-dissatisfied group of clinical control participants obtaining the intervention will be surveyed to identify AN-specific processes. The standardized four-session body exposure intervention using photographs of the own body is adapted from a manualized body image treatment program for computer use. Psychopathology (body dissatisfaction, body image avoidance, body checking, depression, anxiety) is assessed via standardized questionnaires before and after the intervention. During each session, attentional biases regarding one's own body are measured via eye tracking, stress levels are measured via subjective ratings, heart rate variability, as well as salivary cortisol and alpha amylase. Between- and within-subject effects will be assessed. The pilot study aims to identify short-term effects of the intervention on body dissatisfaction and attentional bias, as well as to investigate the potential underlying mechanism of physiological habituation.

## Introduction

Anorexia nervosa (AN) has a lifetime prevalence of between 0.5 and 2%, with a peak age of onset between 13 and 18 years (1). AN is characterized by abnormally low body weight, an intense fear of gaining weight, and a disturbed perception of the own body (2). AN is associated with poor outcomes: Mortality rates are high, and after treatment, less than one half of patients recover, whereas 20% remain chronically ill (3). Specifically, adolescent AN patients show high hospital readmission rates [45% after inpatient treatment (4)].

Body image (BI) is a multifaceted construct including perceptual, affective, cognitive, and behavioral components referring to the own and others' bodies (5). BI disturbances are diagnostic features and stabilizing factors in eating disorders (EDs): patients overestimate their shape and weight (6). Regarding behavioral aspects of BI disturbances, typical features in AN are body checking (repeated measuring and checking of shape and weight) and body avoidance [e.g., covering up the body shape by wearing baggy clothes or avoiding looking at oneself in the mirror (7)]. Despite much fewer studies on adolescents than on adults, a recent systematic review reported more BI disturbances in adolescents with AN and bulimia nervosa (BN) compared to healthy controls (8), similar to findings in adults. Alongside, Legenbauer, Thiemann (5) point out age-dependent differences between children, adolescents, and adults and the need for more studies for the still underresearched group of children and adolescents with eating disorders.

Disturbances of BI in EDs are thought of as a type of cognitive bias, with attentional bias (AB) being a form of biased information processing (9, 10). Evidence suggests that individuals with EDs demonstrate an AB regarding body shape-related stimuli, as examined by, e.g., the Stroop and Dot Probe task (11). ED patients not only show an AB for body-related information (i.e., words), but also for specific body parts (12). Especially, a bias toward negatively rated or most unattractive body parts was found in adolescents with eating disorders (13). This is in contrast to women without an ED diagnosis who showed a balanced attentional pattern regarding self-rated positive and negative body parts of their own body (14). Inducing a temporary AB for self-defined unattractive body parts leads to a significant decrease in body satisfaction in young women without EDs (15). This was confirmed by Tuschen-Caffier and colleagues (16) who found AB to be correlated with body dissatisfaction in women with AN and BN. In a previous study comprising of adolescent ED patients and a healthy control group, we assessed visual attentional processes via eye tracking while watching disease-specific pictures of female bodies (17). While all girls attended more to specific body parts (e.g., hips, upper legs), eating-disordered girls showed an AB toward unclothed body parts, which could be a behavioral marker for overestimation of shape and weight (17).

Based on the relevance of BI disturbances for the maintenance of EDs, it is crucial to target these explicitly (8). Particularly exposure interventions are highly recommended for ED treatment (8, 18, 19). Repeated body exposures in front of a full-length mirror (mirror exposure, ME), with patients wearing tight clothing or underwear to view the body shape and size as best possible, is a commonly used and recommended practice in ED treatment (20). This is applicable to both AN patients approaching a healthy weight and patients with BI disturbances without the diagnosis of AN (20). Former studies have shown ME as a one-time exposure or as part of a more comprehensive treatment program as being effective in the reduction of body dissatisfaction, body checking and body avoidance in adults (19, 21, 22) and adolescents (23) with AN or high body dissatisfaction. Besides ME, other body exposure interventions [e.g., exposure using video recording or virtual reality (24)] have been investigated and evaluated for treating patients with AN in a few studies, with cautiously positive results regarding improvement in BI facets (25). Altogether, evidence for body exposure interventions is still insufficient, especially for adolescent patients—results are mixed, and which patient groups benefit most from which form of body exposure, as well as the mode of action, are unclear (20, 25). Inter alia, modification of the AB and cognitive-affective habituation as along with habituation of physiological processes are discussed as potential underlying mechanisms (20).

Individuals with EDs (mainly investigated for BN) and highly body dissatisfied women show negative cognitive, emotional as well as altered physiological responses during the exposure to their own body [e.g., (26–28)]. For the assessment of stress reactivity, non-invasive measurements are typically used, e.g., heart rate (HR) and heart rate variability [HRV (29)] as sympathetic stress markers. These are affected by emotional stimuli (30) or stress (31). Studies have shown that individuals with AN have a consistently elevated HRV compared to healthy controls, which seems to normalize during recovery (32). Furthermore, salivary alpha amylase (sAA) is a marker of acute stress (33), highly correlates with blood norepinephrine levels (34, 35), and is thereby a further marker of the central sympathetic nervous system [SNS (36, 37)]. Lastly, cortisol, which is a correlate of an activated hypothalamic-pituitary-adrenal (HPA) axis, has been extensively investigated in EDs, showing both altered basal levels and reactivity (38). A study by Monteleone and colleagues (39) showed that individuals with AN exhibit an asymmetry between the HPA axis and the SNS response to a stressor, with the underweight AN group showing a strong cortisol reactivity but an almost completely lacking increase of sAA (comparable to the control groups).

While investigating stress reactivity to body exposure in women with BN who obtained six therapeutic sessions of ME, a decrease of salivary cortisol levels from the initial to the final intervention session was observed; this could be interpreted as habituation of the neuroendocrine stress response (40). Furthermore, a study in women with binge eating disorder found interactions between induced stress and body dissatisfaction during ME by measuring the biological response to social stress via salivary cortisol and sAA (41). Additionally, ED patients showed higher cortisol levels than control participants during a single 40-min body exposure task in a study exploring neuroendocrine (salivary cortisol) and physiological (skin conductance, heart rate) responses in women with EDs, compared to healthy controls. However, no group differences regarding neuroendocrine or physiological reactivity occurred (42). Investigating the effect of a three-session mirror exposure on psychophysiological sympathetic reactivity (HR, HRV, skin conductance) in women with BN vs. healthy controls showed an initial response within the sessions in both groups, but no physiological habituation between the sessions; thereby, the initial response was an unexpected decreasing sympathetic activity which might be explained by the stronger feelings of sadness compared to fear (28). So, until now, exact physiological mechanisms which go along with repeated body exposure in patients with AN or high body dissatisfaction are not yet clarified, with no study, to the authors' knowledge, investigating these in adolescents.

## Objectives

Due to the high association of BI disturbances and the development and maintenance of EDs, including AN, psychotherapy with patients with AN in any setting should include modules treating body image disturbances explicitly (8). A need for new, emerging treatments with a focus on brain-based interventions as well as mechanism-based approaches, which are specifically addressing psychopathology and BI disturbances, have been proclaimed (43, 44). While there is data available on the efficacy of body exposure interventions for reducing BI disturbances in women with EDs and high body dissatisfaction (20, 25), there is a lack of available data regarding the efficacy of body exposures with children and adolescents. Furthermore, different EDs are frequently mixed up in the literature, making it difficult to investigate specific processes, which might differentiate between body dissatisfied individuals with EDs and body dissatisfied individuals without EDs. Also, underlying psychophysiological and neurobiological processes during body exposure are still poorly understood. Therefore, the aim of this study is to investigate the short-term efficacy of a computer-based body exposure intervention for adolescents with AN. This group is being compared to an AN-TAU group and a body dissatisfied clinical control group.

The primary research question is: Is body dissatisfaction in adolescents with AN reduced after completing a four-session computer based body exposure intervention, compared to TAU?

Secondary research questions include: (1) Body checking and avoidance: Can four sessions of a computer based body exposure intervention reduce body checking and body avoidance behavior, compared to TAU? (2) Viewing patterns / attentional bias (AB): Can the AB in terms of focusing more on negatively rated body parts, measured via eye tracking, be reduced by a four-session computer based intervention in adolescents with AN, compared to TAU? Are there AN-specific AB modifications in comparison to a body dissatisfied control group also obtaining the intervention? (3) Stress reactivity: Do four sessions of body exposure modify stress reactivity in terms of a physiological habituation, measured via salivary cortisol, sAA, HR and HRV? Are there AN-specific effects in physiological habituation in comparison to a body dissatisfied clinical control group?

## Materials and Methods

### Context (FRAnconian Longitudinal Study of Anorexia Nervosa in Adolescents)

The body exposure intervention described in this study protocol is part of the bigger FRALANA study (FRAnconian Longitudinal study of Anorexia Nervosa in Adolescents), which is being carried out at the Division of Child and Adolescent Mental Health, University Hospital Erlangen. The FRALANA study aims to broaden the knowledge of AN pathogenesis and the understanding of underlying mechanisms of AN treatment in children and adolescents, using a multi-level approach. The FRALANA study consists of a basic and an intervention module. For the basic module, data is collected from three groups of participants (AN group, depressive control group, healthy control group), across three different points in time during and after inpatient treatment. The intervention module investigates short term effects of a computer based body exposure intervention and is described in detail in the following paragraphs. Ethical approval for the FRALANA study was granted by the local ethics committee of the Medical Faculty at the Friedrich-Alexander-University Erlangen-Nuremberg (ID: 257_20 B). The study is registered with the German Clinical Trials Register (Deutsches Register Klinischer Studien; DRKS), ID number DRKS00024675. The study is conducted in accordance with the Declaration of Helsinki. All adolescents and their parents, or young women, respectively, have to give informed consent before participating.

### Design

**Figure 1:**
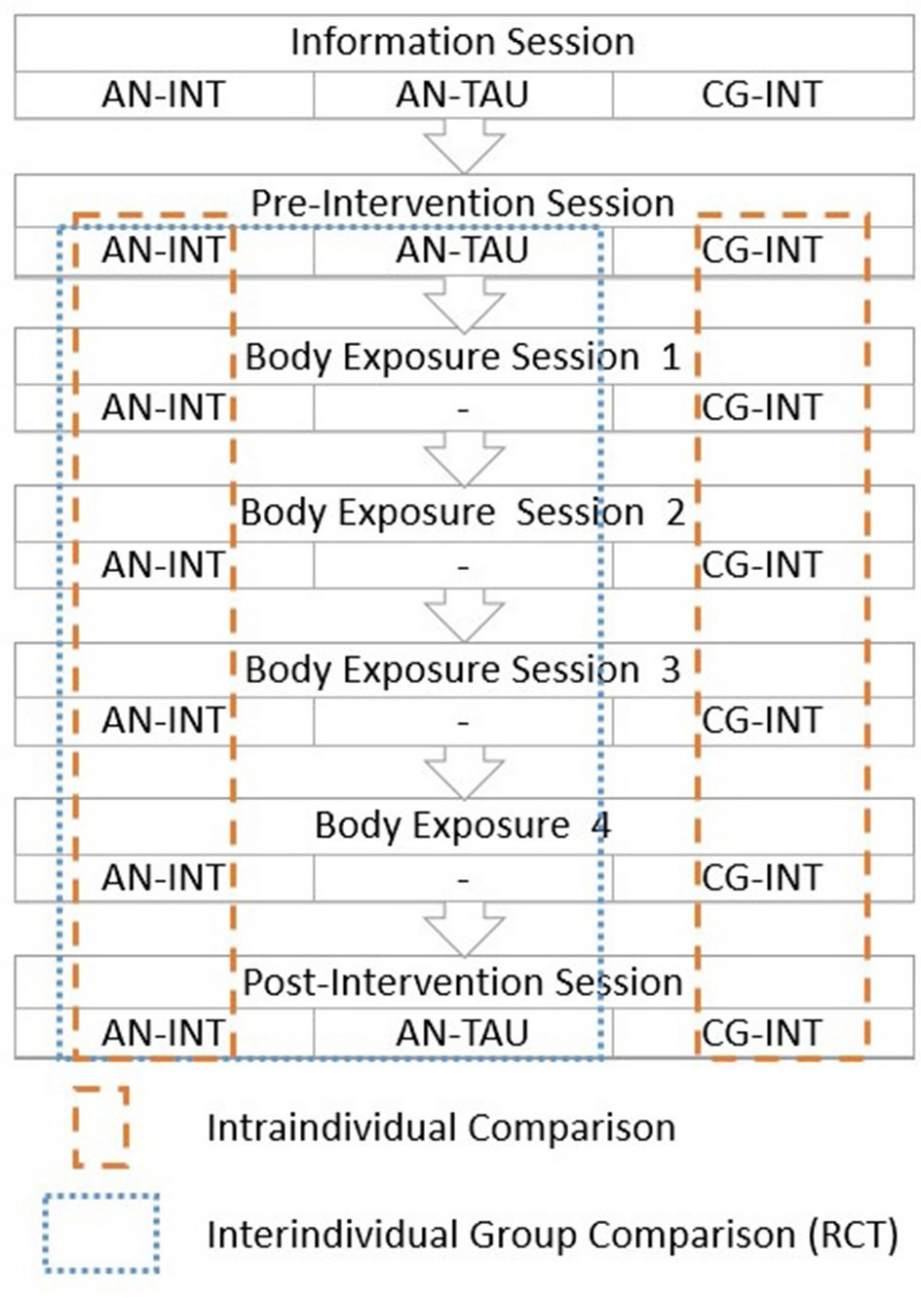
Study Design. The figure shows the sessions which the different groups complete (AN-INT, Anorexia Nervosa Group-Intervention; AN-TAU, Anorexia Nervosa Group-Treatment As Usual; CG-INT, Body Dissatisfied Control Group-Intervention) and how the groups will subsequently be compared. Within the RCT, adolescents with AN obtaining the intervention (AN-INT) vs. treatment as usual (AN-TAU) will be compared to each other. In a transdiagnostic approach, a body-dissatisfied group of clinical control participants (CG-INT) obtaining the intervention will be compared to AN-INT in order to identify AN-specific processes. Intraindividual processes will be analyzed for AN-INT and CG-INT.

Within a randomized controlled trial (RCT) female adolescents and young women with AN are either receiving four sessions of a computer based body exposure intervention (AN-INT) or treatment as usual (AN-TAU). Additionally, a highly body dissatisfied group of clinical control participants (CG-INT) are undergoing the intervention to identify AN-specific processes of the intervention. The study design, including study groups, time points and methods of assessment, is shown in Figure 1. The study includes intraindividual (pre- vs. post-intervention; modifications throughout the four exposure sessions) and interindividual (AN-INT vs. AN-TAU; AN-INT vs. CG-INT) research. The TAU group only completes the pre- and post-intervention sessions with assessment of psychopathology and a recording of gaze patterns.

### Target/Study Population

Participants must be between 10 and 21 years of age and of female gender. The following groups will be included in the study:

(1) AN patients (*AN*): Diagnosis of AN according to ICD-10 criteria (ICD-10: F50.0 or F50.1) diagnosed by an experienced (child) psychiatrist or psychologist using the “Kinder-DIPS” diagnostic interview (45, 46) in adolescents and the Structured Diagnostic Interview for Mental Disorders [DIPS; (47)] in adults;

(2) Body dissatisfied control group (*CG*): Patients with a high level of body dissatisfaction (according to the EDI-2 subscale “Body dissatisfaction“ [highest quartile = >75th percentile] and clinical judgment of an experienced (child) psychiatrist or psychologist).

At study inclusion, all children and adolescents are required to have a body weight > 10th BMI age percentile, or a BMI > 18.5 kg/m^2^ for young women, respectively, corresponding to a weight range above the classification of being underweight to avoid habituation at very low weight in AN participants. Participants will be recruited during inpatient, day clinic, or outpatient treatment at the Division of Child and Adolescent Mental Health and the Department of Psychosomatic Medicine and Psychotherapy. Antidepressant or antipsychotic medication is not an exclusion criterion; however, acute psychotic symptoms, use of illegal substances, medication with sedating effects, chronic somatic diseases, intellectual disability (IQ <85) and insufficient understanding of the German language are exclusion criteria.

Until now and to the best of our knowledge, no study using a comparable computer based body exposure intervention using photographs of the own body could be identified. Because of this, sample size calculation for the current pilot study was performed in accordance with former studies assessing effects of mirror (body) exposure in adolescents and adults with EDs (23, 42), as well as fear-stimulus exposure in phobic adolescents (48), reporting mostly medium effect sizes for body avoidance (body) anxiety and negative emotions. According to sample size calculations [G^*^Power; (49)] based on default values (alpha error =.05, power =.80) and the assumption that effect sizes for the RCT (AN-INT vs. AN-TAU) are expected to be larger than for the comparison of AN-INT vs. CG-INT, both taking part in the same intervention, at least 23 participants are required for the intervention groups (AN-INT, CG-INT) and at least 15 participants are required for AN-TAU. This is calculated based on a 2 × 2-mixed design (RCT; between-subject factor: AN-INT vs. AN-TAU; repeated measure within-subject factor: pre, post) and a 2 × 4-mixed design (AN-INT vs. CG-INT; repeated measure within-subject factor: 4 exposure sessions), respectively.

### Parameters

Psychopathology is assessed through the completion of multiple questionnaires: Eating disorder psychopathology and body dissatisfaction are assessed by administering the Eating Disorder Inventory-2 [EDI-2 (50), German version (51)], body image avoidance behavior via the Body Image Avoidance Questionnaire [BIAQ (52), German version (53)], and body checking behavior by the Body Checking Questionnaire [BCQ (54), German version (55)]. To assess relevant comorbid symptoms, we are also administering the Beck Depression Inventory-Revised [BDI-II (56), German version (57)] to test for depressive symptoms, and the Fear Survey Schedule for Children-Revised [FSSC-R (58), German version (59)] to test for symptoms of anxiety. Additionally, body parts are rated for self-perceived attractiveness on a seven point Likert-type scale (ranging from very negative to very positive) and participants are asked to list their three most disliked body parts. In order to control for a potential attention deficit we also administer an attention test [d2 test (60).

Attentional bias is assessed by recording gaze patterns during body exposure via the Eyegaze Analysis System^TM^ (Interactive Minds, Dresden, Germany), which is an infrared video-based binocular tracking system (17). In particular, the subsequent analysis is focused on assessing the fixation patterns (duration, frequency) on the previously three most negatively rated body parts, which are defined as regions of interest (ROI). A fixation is counted if the participant's gaze is focused on a specific area for 100 ms or more. Total fixation time for each ROI is calculated and corrected for its size relative to the picture size (17).

Stress levels and stress reactivity are assessed through multiple measures. Firstly, subjective emotion ratings (anxiety, anger, disgust, stress, and sadness) are obtained on a ten-point Likert scale before, during, directly after, and 30 min after each exposure session. Secondly, participants' cardiac activity is monitored during the exposure sessions using a Scosche^®^ Rhythm 24 Heart Rate monitor. Raw data is further processed using Python (https://www.python.org/). RR intervals (RRI) will be detected by a peak-detection algorithm and outliers will be removed using implemented python libraries (e.g., hrv-analysis 1.0.4 package). Instantaneous heart rate (IHR), as a measurement of heart rate variability (HRV), will be derived following IHR$=\frac{60000}{RRI}$, where RRI are measured in milliseconds (61). Changes in HR/IHR and RRI in relation to the number of exposure sessions, i.e., changes over the course of the four exposure sessions, will be analyzed. Thirdly, saliva samples are taken before, during (10 min into the exposure), directly after and 30 min after the body exposure using Salivettes^®^ (Sarstedt, Nümbrecht, Germany). Cortisol, as well as sAA, levels are subsequently analyzed in the laboratory. To do this, saliva samples are stored at −20°C until processing. When processing begins, the samples are thawed and centrifuged at 2,000 × g and 20°C for 10 min. To analyze salivary free cortisol, a commercial chemiluminescence immunoassay (CLIA; IBL Hamburg, Germany) is used, following the manufacturer's instructions. Similarly, to measure sAA, a quantitative enzyme kinetic method is employed, following a standardized protocol.

### Procedure

For each participant, the whole intervention cycle consists of six sessions and will take place over ~2.5 weeks. After a pre-intervention session, the intervention itself comprises of four body exposure sessions (with a duration of ~45 min each), followed by a diagnostic post-intervention session (with a duration of about 10 min). These are described in more detail below. The body exposure intervention is set upon the body exposure module of the manualized body image treatment program by Vocks and Legenbauer (62). This treatment program has been shown to be a successful technique for improving the outcome of ED treatment in adults (63). The procedure has been adapted for the current study in terms of participants being exposed to photographs of the own body via a computer, instead of using a mirror, and the manualized instructions for each exposure session being transformed into an audio file to ensure a standardized procedure. Throughout all body exposure sessions each participant is guided and accompanied by a permanently assigned female attendant of the research team.

#### Pre-intervention Session

The pre-intervention session starts with the completion of the questionnaires, the attractiveness-ratings of the body parts and the attention test. Furthermore, some theoretical background regarding exposure therapy is explained (psychoeducation). Afterward, two photographs of the participant wearing standardized sportswear (black sports bra and tight shorts; one picture each in frontal and lateral view) will be taken. Lastly, the participant receives an introduction to the eye tracking methodology and the saliva collection technique. For the AN-TAU group, an assessment of AB is taken during this session.

#### Body Exposure Sessions

**Figure 2:**
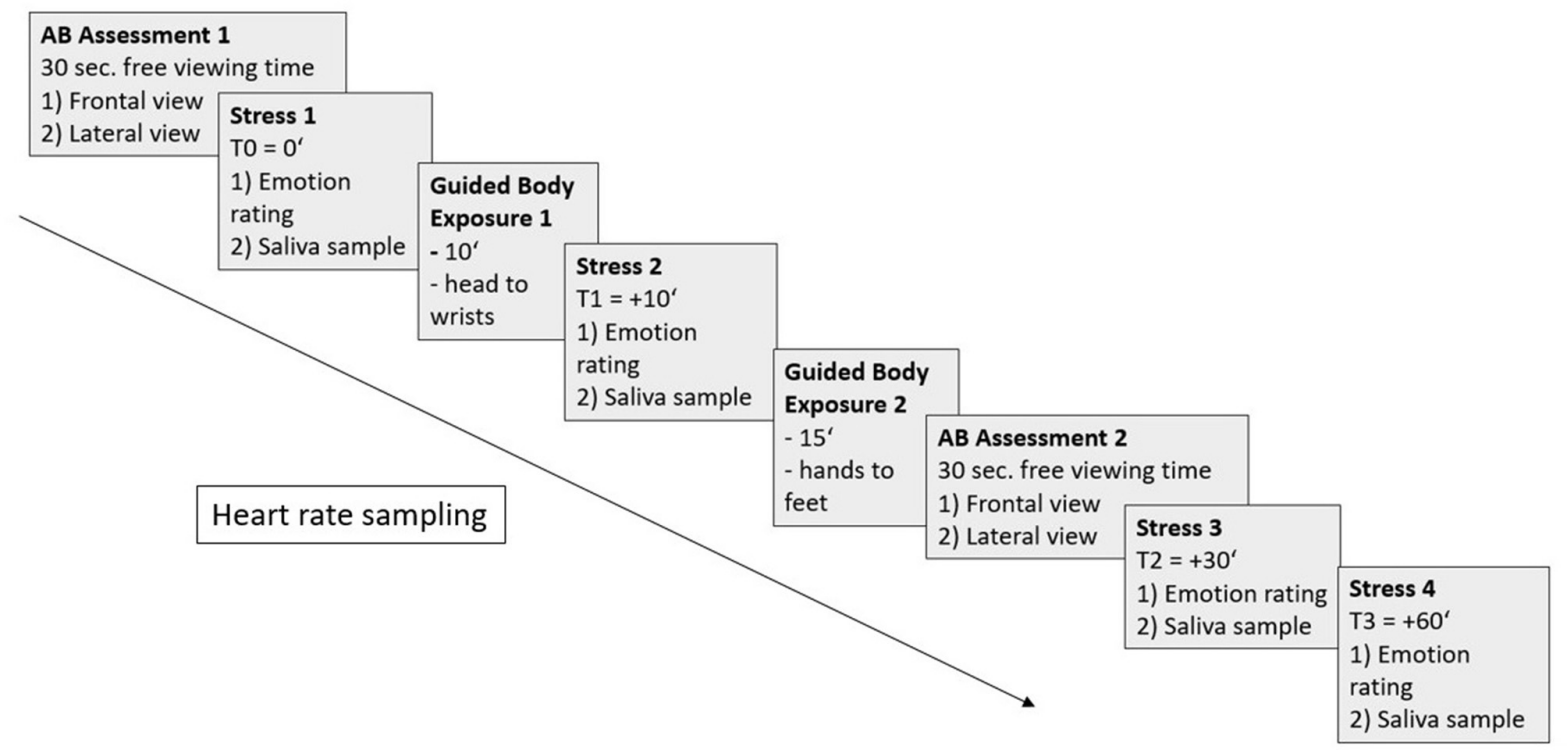
Standardized procedure of an exposure session with attentional bias (AB) assessments (1,2) for each frontal and lateral view (30 s each), stress measurements (1-4) via emotion ratings and saliva samples, guided body exposure times (1,2), and heart rate sampling (from AB Assessment 1 to Stress 3).

The four computer based body exposure sessions are identical and involve a guided exposure to the previously taken photographs of the participant's own body on the computer screen. Standardized instructions are played to the participants via audio file and reactions and short descriptions of their body parts will be supervised by the study attendant who is present during the sessions. In the beginning and at the end of each session, there is a 30 s period of free viewing time for each the frontal and lateral photograph. During this time, participants are viewing the pictures without any instruction and eye movements are recorded in order to measure AB. The audio file guides participants in viewing their own bodies on the computer screen for an overall duration of about 30 min. The body exposure follows an adapted version of the manualized treatment program by Vocks and Legenbauer (62), in which participants are guided to look at 12 body parts following a fixed sequence. To assess stress reactivity, four saliva samples are taken before, during directly after and 30 min after the end of the body exposure with emotions being rated at the same times. Heart rate variability will be assessed continuously throughout each session via heart rate monitor. The standardized procedure of a body exposure session is shown in Figure 2.

#### Post-intervention Session

During the post-intervention session, participants are again asked to complete questionnaires, including the EDI-2, the BIAQ, the BCQ, and the attractiveness ratings of the body parts. For the AN-TAU group, AB is assessed again during this session.

### Data Analysis

Research questions will be analyzed in repeated measures (rm)-AN(C)OVAs, considering within-subject and between-subject factors and interpreting—depending on the respective hypothesis—main and/or interaction effects. To test intervention effects on body dissatisfaction, body control and body avoidance, as well as AB comparing AN-INT vs. AN-TAU within the RCT, a 2 × 2-rm-AN(C)OVA (between-subject factor = group: AN-INT vs. AN-TAU; within-subject factor = time: pre, post) will be conducted. To assess modifications within and throughout the body exposure sessions regarding AB, emotion ratings, HR, HRV, as well as cortisol and sAA reactivity slope, a 2 × 4-rm-AN(C)OVA (between-subject factor = group: AN-INT vs. CG-INT; within-subject factor = time: 4 exposure sessions) will be conducted. Potential confounders are derived from a theoretical point of view (e.g., age, depression) and will be included as covariates in the statistical analyses (ANCOVAs). Specific test requirements will be tested prior to performing the full analysis. In case of non-adherence to the test requirements (i.e., normal distribution and variance homogeneity), the outcome measures will be logarithmized and additional non-parametric tests will be calculated. Data will be analyzed using IBM SPSS Statistics software. Significance level will be set at *p* =.05 and effect sizes will be interpreted. Effect sizes for AN(C)OVA results were computed as partial η^2^ ($\eta_{\text{p}}^{2}$), with values $\eta_{\text{p}}^{2}$ <0.06 interpreted as small,.06 ≤ $\eta_{\text{p}}^{2}$ <0.14 as medium and $\eta_{\text{p}}^{2}$ ≥ 0.14 as large effects (64). Correction for multiple testing will be applied if necessary.

## Strengths and Expected Outcomes

The multi-level approach of this study allows for both an objective and a subjective evaluation of the short-term effects of the proposed computer based body exposure intervention. It will give new insights into the effects and underlying processes of the underresearched field of body exposure in adolescents with AN. The collected data will be assessing different aspects of body dissatisfaction and general psychopathology by employing questionnaires, measuring AB, utilizing emotion ratings, recording HR and HRV, as well as determining salivary cortisol and sAA levels, throughout the exposure sessions. Besides comparing the pre-post intervention effect with TAU within an RCT, this study will also investigate how the processes that underlie body exposure change throughout the intervention and if there are AN-specific modifications in comparison to adolescents with high body dissatisfaction (CG-INT). Corresponding with the current literature regarding body exposure interventions, especially for adolescents with AN, we expect psychopathology (body dissatisfaction, body image avoidance, body checking) to be reduced at the end of the intervention compared to the beginning of the intervention and with TAU and the AB to weaken (shown by spending less time looking at the negatively rated body parts). Furthermore, we expect a physiological habituation, both within and across sessions, with a decrease in emotion ratings, HR and neuroendocrine stress levels. To put it in a nutshell, results of this pilot study will give new insights into the effectiveness of and the processes underlying body exposure interventions in children, adolescents, and young women. Despite a relatively small sample size, a comprehensive in-depth analysis, implemented by multi-level assessment, will be carried out, thereby expanding the so far weak literature on mechanisms of BI disturbances and specific interventions for the abovementioned patient groups. The results of the current pilot study will be used for the design of a subsequent larger, multi-center intervention study with adolescents with AN, helping e.g., to identify modifiable BI and psychopathologic outcome measures, to select relevant and modifiable stress markers, and to provide orientation toward expectable effect sizes.

## Author Contributions

VS, OK, GM, and SH initiated and designed the study. VS, LS, and SH wrote and prepared the manuscript. All authors reviewed the manuscript.

## Conflict of Interest

The authors declare that the research was conducted in the absence of any commercial or financial relationships that could be construed as a potential conflict of interest.

## Publisher's Note

All claims expressed in this article are solely those of the authors and do not necessarily represent those of their affiliated organizations, or those of the publisher, the editors and the reviewers. Any product that may be evaluated in this article, or claim that may be made by its manufacturer, is not guaranteed or endorsed by the publisher.
