# Corrigendum: Computer based body exposure in adolescents with anorexia nervosa: a study protocol

**DOI:** 10.3389/fpsyt.2025.1576099

**Published:** 2025-04-22

**Authors:** Valeska Stonawski, Lena Sasse, Gunther Moll, Oliver Kratz, Stefanie Horndasch

**Affiliations:** Department of Child and Adolescent Psychiatry, Faculty of Medicine, Friedrich-Alexander-University Erlangen-Nürnberg, Erlangen, Germany

**Keywords:** anorexia nervosa, body dissatisfaction, body exposure, eye tracking, attentional bias, adolescence, stress reactivity, randomized controlled trial (RCT)

In the published article, an **Acknowledgments** section was mistakenly not included in the publication. This section has now been added:

“**Acknowledgments**

This paper is part of the fulfillment of obtaining a doctoral degree for Lena Sasse.”

The authors apologize for this error and state that this does not change the scientific conclusions of the article in any way. The original article has been updated.

